# Lamina Associated Polypeptide 1 (LAP1) Interactome and Its Functional Features

**DOI:** 10.3390/membranes6010008

**Published:** 2016-01-15

**Authors:** Joana B. Serrano, Odete A. B. da Cruz e Silva, Sandra Rebelo

**Affiliations:** Neuroscience and Signalling Laboratory, Department of Medical Sciences, Institute of Biomedicine—iBiMED, University of Aveiro, 3810-193 Aveiro, Portugal; jmbs@ua.pt (J.B.S.); odetecs@ua.pt (O.A.B.C.S.)

**Keywords:** Lamina associated polypeptide, nuclear envelope, Inner nuclear membrane, interactors, network, database, Cytoscape, GeneMANIA, GO terms enrichment analysis, Ingenuity pathway analysis

## Abstract

Lamina-associated polypeptide 1 (LAP1) is a type II transmembrane protein of the inner nuclear membrane encoded by the human gene *TOR1AIP1*. LAP1 is involved in maintaining the nuclear envelope structure and appears be involved in the positioning of lamins and chromatin. To date, LAP1’s precise function has not been fully elucidated but analysis of its interacting proteins will permit unraveling putative associations to specific cellular pathways and cellular processes. By assessing public databases it was possible to identify the LAP1 interactome, and this was curated. In total, 41 interactions were identified. Several functionally relevant proteins, such as TRF2, TERF2IP, RIF1, ATM, MAD2L1 and MAD2L1BP were identified and these support the putative functions proposed for LAP1. Furthermore, by making use of the Ingenuity Pathways Analysis tool and submitting the LAP1 interactors, the top two canonical pathways were “Telomerase signalling” and “Telomere Extension by Telomerase” and the top functions “Cell Morphology”, “Cellular Assembly and Organization” and “DNA Replication, Recombination, and Repair”. Once again, putative LAP1 functions are reinforced but novel functions are emerging.

## 1. Introduction

The eukaryotic nucleus is a complex organelle enclosed by a highly organized double membrane, the nuclear envelope (NE). The NE separates the nucleus from the cytoplasm and is essentially composed by the inner nuclear membrane (INM), the outer nuclear membrane (ONM), the nuclear pore complexes (NPCs) and nuclear lamina. The INM and ONM are separated by the perinuclear space of 40–50 nm of diameter and are crossed and therefore connected at the NPCs. The perinuclear space is continuous with the lumen of the rough endoplasmic reticulum (RER) and the ONM is continuous with the rough endoplasmic reticulum membrane that comprises the ribosomes. The lamins, which are type V intermediate filament proteins exclusively found in the nucleus and associated with INM proteins, can be found in the nuclear lamina. The NE and NE proteins have received more attention in the last few years and there is increasing evidence that the NE is responsible for integrating many cellular functions, including chromatin organization, signalling pathways, transcription regulation and cytoskeletal organization [1,2,3].

The nuclear membranes are considered an interconnected membrane system associated with the RER that comprises a specific group of proteins that are specifically enriched in the INM and ONM, but not in the RER. Of these, approximately 80 transmembrane proteins are concentrated in the INM [4] and a significantly lower number is concentrated in the ONM. Some of the INM proteins remain uncharacterized, but others were found to interact with lamins and/or chromatin. One of the first lamina associated proteins identified was lamina-associated polypeptide 1 (LAP1) [5] which is a type II transmembrane protein of the inner nuclear membrane, encoded by the human gene *TOR1AIP1*. In rats, three LAP1 isoforms were described and are derived by alternative RNA splicing, these are LAP1A, LAP1B and LAP1C with molecular weights of 75, 68 and 55 KDa, respectively [5,6]. In humans, the LAP1B isoform was previously identified by Kondo *et al.* [7] and a novel human isoform, the LAP1C, was recently identified. This new isoform is N-terminally truncated, with a molecular weight of approximately 55 KDa contrasting with the 68 KDa of the LAP1B [8]. The function of LAP1 remains poorly understood. However, several LAP1 binding partners have been identified as is the case with lamins (directly binding) and chromosomes (indirectly binding) [9]. Therefore it is assumed that LAP1 might be involved in the positioning of lamins and chromatin in close proximity to the NE, thereby contributing to the maintenance of NE structure [6,10]. Another important LAP1 binding protein is torsinA, which is the central protein in DYT1 dystonia [11]. A mutation of a single glutamic acid within torsinA (∆E-torsinA) is responsible for DYT1 dystonia, a dominantly inherited neurological and movement disorder characterized by prolonged involuntary twisting movements [12]. Interestingly, while the wild type torsinA is localized in both RER and the perinuclear space, the mutated torsinA (∆E-torsinA; pathogenic variant) preferentially concentrates in the perinuclear space [13,14]. Of note, torsinA variants that bind more efficiently to LAP1 do not hydrolyze ATP. Furthermore, LAP1 has been shown to bind torsinA and to activate its ATPase activity [15,16]. Recently, LAP1 was found to interact with another INM protein, namely emerin [17], the latter is associated with the X-linked Emery-Dreifuss muscular dystrophy [18]. The interaction of these two INM proteins is mediated via their nucleoplasmic domain, whereby emerin binds to LAP1 residues 1-330 [17]. We recently reported that the human LAP1B binds to protein phosphatase 1 (PP1) in the nucleoplasm domain and that it is dephosphorylated by this phosphatase [19]. Furthermore, five different LAP1 phosphorylated residues were identified: Ser143, Ser216, Thr221, Ser306 and Ser310. From these, it was possible to establish that only Ser306 and Ser310 are dephosphorylated by PP1 [8].

Recently, two mutations were identified in the *TOR1AIP1* gene, that were directly associated with disease conditions. The first *TOR1AIP1* mutation (“Turkish” mutation) reported, affects three members of a Turkish family with an autosomal recessive limb-girdle muscular dystrophy with joint contractures. It was predicted that this c.186delG mutation, truncates the 584 amino acid LAP1B protein to an apparently non-functional protein, only 83 amino acids long [20]. Consequently, expression of LAP1B was absent in the skeletal muscle fibers of these patients. The authors also showed, by ultrastructural examination of the muscle fibers, that patients had intact sarcomeric organization but clearly evident alterations of the nuclear envelope including nuclear fragmentation. Of note, this study places LAP1B as a pivotal protein associated with striatal muscle function and increases the number of genes associated with nuclear envelopathies. A second *TOR1AIP1* mutation (“Maroccan” mutation) was reported in a boy, born from consanguineous healthy parents, who developed rapidly progressing dystonia, progressive cerebellar atrophy, and dilated cardiomyopathy. Upon whole exome sequencing a homozygous missense mutation in *TOR1AIP1* was identified, resulting in a highly conserved glutamic acid change to alanine at amino acid 482 [21]. The *in vivo* studies performed by the authors indicated that the patients revealed reduced expression levels of LAP1 and its misallocation and aggregation in the endoplasmic reticulum [21]. Of note, both LAP1 human isoforms (LAP1B and C) are affected by this mutation. These two mutations strengthen functional association of LAP1 with DYT1 dystonia (in neurons) and muscular dystrophy (skeletal muscle) [22].

To date the precise LAP1 function is not fully elucidated. However a significant amount of work has been associated with the identification and functional characterization of LAP1 interactions, particularly with lamins, torsinA, emerin and PP1. The identification of additional LAP1 binding proteins and their association to specific cellular pathways and cellular functions are therefore crucial to understanding the precise physiological roles of LAP1. The work here described proposes novel LAP1 functions, through the bioinformatic analysis of the protein’s interactors.

## 2. Results and Discussion

**Figure 1:**
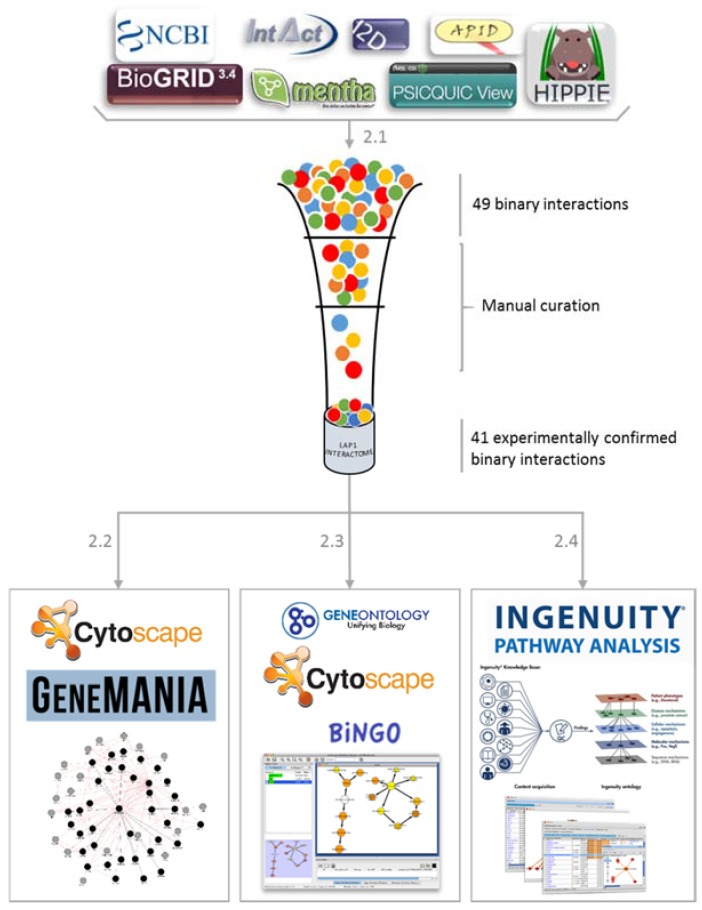
Methods overview. Schematic representation of the workflow; the steps necessary to integrate the Network of LAP1 integrators are depicted.

In order to determine LAP1 interacting proteins the approach summarized in Figure 1 was used. Briefly, binary interactions with LAP1 were collected and manually curated to obtain the working dataset of LAP1 interactors of (step 2.1, Figure 1). Subsequent integration of the interactions among the listed interactors was performed with GeneMANIA, resulting in a more complete prospective network (step 2.2, Figure 1). GO and BiNGO enrichment analysis assisted in ascertaining the most prevalent biological processes and the cellular distribution of the interactors (step 2.3, Figure 1). Finally, by resorting to Ingenuity Pathway Analysis the physiological and functional relevance of the selected proteins were explored (step 2.4, Figure 1).

### 2.1. LAP1 Interactor’s Network

**Figure 2:**
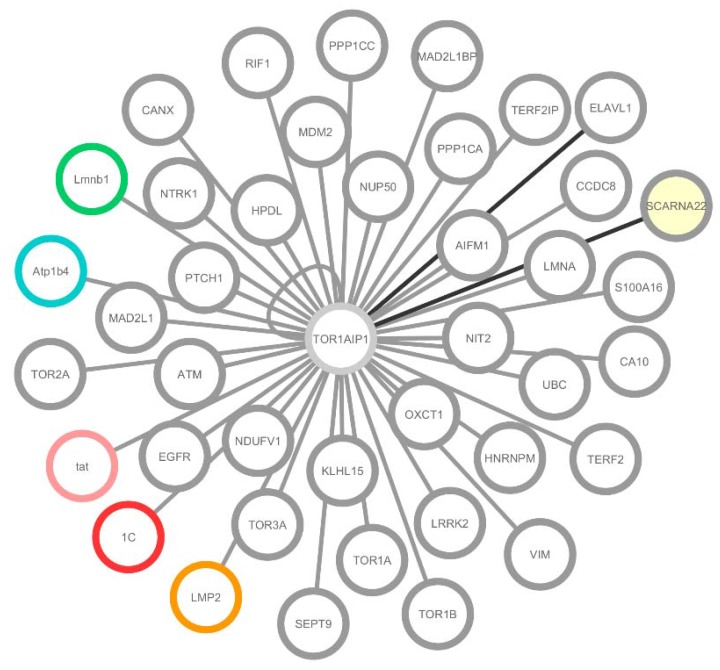
LAP1 interactome built using Cytoscape 3.2.1 [1]. Of these interactions, 36 were verified in human (**grey**); one in mouse and rat (**blue**); one only in rat (**green**); and three between human LAP1 and viral proteins (**pink** for HIV-1, red for HRSVA and orange for HHV-4). All the white nodes represent proteins, while RNA is denoted with yellow filling. Grey edges signify protein-protein interaction, while protein-RNA or RNA-RNA interactions are coloured with black edges. LAP1 is also able to form dimers, which is represented by a self-binding edge.

Having curated information retrieved from the online databases, all the experimentally tested interactions (Table 1) were loaded into Cytoscape 3.2.1 [23] in order to manually build the network of LAP1 interactors (Figure 2). Of these interactions, 36 were verified in human (grey), one in mouse and rat (blue), one only in rat (green) and three between human LAP1 and viral proteins (pink for HIV-1, red for HRSVA and orange for HHV-4). All the white nodes represent proteins, while RNA is denoted as yellow nodes. Grey edges signify protein-protein interaction, while protein-RNA or RNA-RNA interactions are coloured in black (Figure 2).

Throughout the construction of this particular dataset one might consider the fact that every experimental method used in the study of protein-protein interactions has its drawbacks. In Table 1, the techniques included two-hybrid, affinity capture-MS/WB, co-fractionation and reconstituted complex. These techniques give completely different information but together they enable the identification of LAP1 putative biological functions. Two-hybrid and reconstructing complexes indicate direct protein-protein interactions, even if their biological relevance is questionable. Conversely, affinity capture-MS/WB and co-fractionation represent interactions that could be indirectly occurring in a complex. Consequently, one has to be critical when considering the interactions that are indicated in Table 1.

The methodology used for validation of the interaction and the number of times it was validated can strengthen the proposed interaction. More relevance should be given to the interactions proven multiple times through different techniques. For instance, the interaction between LAP1 and TorsinA has been validated in nine different publications by affinity capture-MS/WB and reconstituted complex methods. Additionally, the LAP1-TorsinA complex has been investigated several times [22,24] as well as the LAP1-Lamin A complex, which is associated to the maintenance of the nuclear envelope structure. Conversely, the Nuclear pore complex protein, Nup50, has been described with only one publication, using co-fractionation protocols but the biological function was not investigated. Considering these examples, there is stronger evidence for TorsinA and Lamin A as LAP1 interactors than for Nup50.

The bioinformatic analysis retrieved two RNA integrating interactions with LAP1. The first of these is ELAVL1, an RNA-binding protein that regulates the stability and translation of numerous mRNAs encoding proteins that respond to stress or proliferation [25]. ELAV-like protein 1 physically interacts with *TOR1AIP1* mRNA (Figure 2), possibly promoting stability or influencing translation, in response to environmental changes [25]. The other RNA interaction described was with WHSC1, which is a histone methyltransferase (also known as MMSET) that has a sequence for ACA11 within an intron of its gene [26]. ACA11 is an orphan box H/ACA class small nuclear RNA (snoRNA) that is localized to the nucleoli and integrates a new snRNP (small nuclear ribonucleoprotein) complex that is involved in post-splicing intron complexes [26]. This snRNP is proposed to target other snoRNAs intermediates, in particular, snoRNAs hosted within ribosomal protein (RB) genes and to bind various nucleolar proteins associated with the regulation of RNA processing [26]. Functionally, ACA11 was found to suppress oxidative stress, confer resistance to chemotherapy and increase the proliferation in multiple myeloma cells. In the work of Chu and colleagues [26], LAP1 was found to bind this snoRNA, where both share binary interactions with HNRNPM, VIM, LMNA and LMNB1 (Figure 2) [26]. Given the absence of additional functional studies on ACA11, the meaning of its associations with LAP1 and its interactors remains unclear. However, the emerging hypothesis is that LAP1 might be associated with transcription regulation through the interaction with ACA11 and HNRNPM (Figure 2).

Moreover, a subset of viral proteins that integrate the LAP1 network, namely tat, 1C and LMP2 (Figure 2) was also identified. Tat is a nuclear regulatory protein crucial for HIV-1 replication that also coordinates HIV-1 provirus transcriptional regulation [27]. Alternatively, RNA viruses like HRSV have limited coding capacity, and their proteins often possess multiple functional domains with the ability to interact either with viral or cellular proteins [28]. This is the case for 1C, which binds various human proteins that are associated with transcriptional and cell cycle regulation [28]. Lastly, the Epstein-Barr virus LMP2B isoform co-localizes with LMP2A in perinuclear regions in transiently transfected cells [29], however LMP2 function in the NE remains unclear.

Due to the significant role of the NE as a cellular barrier, multiple viral organisms have developed the capability to modulate their permeability in order to infect the host cell (reviewed in [30]). Viruses can modulate NE permeability for different reasons. Some viruses disrupt NE in order to transport the viral genome into the nucleus for replication (HIV), while others cause NE disruption during nuclear egress of newly assembled capsids (HHV-4) (reviewed in [30]). In addition, many viruses modulate NE permeability either to destabilize compartmentalization of host proteins or to inhibit the nuclear transport of host antiviral response proteins (reviewed in [30]).

**Table 1:** LAP1 interactors obtained from online databases after validating curated interactions via consulting respective publications.

Gene	Protein	Uniprot Accession Number	Species	Interaction Detection Method	References
***1C***	Non-structural protein 1	P04544	*TOR1AIP1 Hs—1C HRSVA*	Affinity Capture-MS	[28]
***AIFM1***	Apoptosis-inducing factor 1, mitochondrial	O95831	*Homo sapiens*	Affinity Capture-MS	[31]
***ATM***	Serine-protein kinase ATM	Q13315	*Homo sapiens*	Affinity Capture-MS	[32]
***Atp1b4***	Protein ATP1B4	Q99ME6	*Mus musculus*	Two-hybrid	[33]
		Q9R193	*Rattus norvegicus*	Affinity Capture-WB	
***CA10***	Carbonic anhydrase-related protein 10	Q9NS85	*Homo sapiens*	Affinity Capture-MS	[34]
***CANX***	Calnexin	P35564	*Tor1aip1 Mm—CANX Hs*	Affinity Capture-MS	[35]
***CCDC8***	Coiled-coil domain-containing protein 8	Q9H0W5	*Homo sapiens*	Affinity Capture-MS	[36]
***EGFR***	Epidermal growth factor receptor	P00533	*Homo sapiens*	Affinity Capture-MS	[37]
*ELAVL1* *	ELAV-like protein 1	Q15717	*Homo sapiens*	Affinity Capture-RNA	[25]
***HNRNPM***	Heterogeneous nuclear ribonucleoprotein M	P52272	*Homo sapiens*	Co-fractionation	[38]
***HPDL***	4-hydroxyphenylpyruvate dioxygenase-like protein	Q96IR7	*Homo sapiens*	Co-fractionation	[38]
***KLHL15***	Kelch-like protein 15	Q96M94	*Homo sapiens*	Affinity Capture-MS	[31]
***LMNA***	Prelamin-A/C	P48678	*Mus musculus*	Affinity Capture-MS	[39]
		P02545	*Homo sapiens*	Two-hybrid	[40]
			*Homo sapiens*	Reconstituted Complex	[9]
			*Homo sapiens*	Proximity Label-MS	[41]
***Lmnb1***	Lamin-B1	P70615	*Rattus norvegicus*	Affinity Capture-WB	[42]
***LMP2***	Latent membrane protein 2	Q1HVJ2	*TOR1AIP1 Hs—LMP2 HHV-4*	Affinity Capture-MS	[43]
***LRRK2***	Leucine-rich repeat serine/threonine-protein kinase 2	Q5S007	*Homo sapiens*	Affinity Capture-MS	[44]
***Mad2l1***	MAD2 mitotic arrest deficient-like 1	Q9Z1B5	*TOR1AIP1 Hs—Mad2l1 Mm*	Affinity Capture-MS	[35]
***MAD2L1BP***	MAD2 mitotic arrest deficient-like 1	Q15013	*Homo sapiens*	Affinity Capture-MS	[31]
***MDM2***	E3 ubiquitin-protein ligase Mdm2	Q00987	*Homo sapiens*	Affinity Capture-MS	[45]
***NDUFV1***	NADH dehydrogenase [ubiquinone] flavoprotein 1, mitochondrial	P49821	*Homo sapiens*	Co-fractionation	[38]
***NIT2***	Omega-amidase NIT2	Q9NQR4	*Homo sapiens*	Co-fractionation	[38]
***NTRK1***	High affinity nerve growth factor receptor	P04629	*Homo sapiens*	Affinity Capture-MS	[46]
***NUP50***	Nuclear pore complex protein Nup50	Q9UKX7	*Homo sapiens*	Co-fractionation	[38]
***OXCT1***	Succinyl-CoA:3-ketoacid coenzyme A transferase 1, mitochondrial	P55809	*Homo sapiens*	Co-fractionation	[38]
***PPP1CA***	Serine/threonine-protein phosphatase PP1-alpha catalytic subunit	P62136	*Homo sapiens*	Affinity Capture-WB	[47]
				Two-hybrid	[48]
***PPP1CC***	Serine/threonine-protein phosphatase PP1-gamma catalytic subunit	P36873	*Homo sapiens*	Affinity Capture-WB	[47]
				Two-hybrid	[49]
***PTCH1***	Protein patched homolog 1	Q13635	*Homo sapiens*	Affinity Capture-MS	[34]
***RIF1***	Telomere-associated protein RIF1	Q5UIP0	*Homo sapiens*	Co-fractionation	[38]
*SCARNA22* **	Small Cajal body-specific RNA 22	Gene ID: 677770	*Homo sapiens*	Affinity Capture-RNA	[26]
***S100A16***	Protein S100-A16	Q96FQ6	*Homo sapiens*	Co-fractionation	[38]
***SEPT9***	Septin-9	Q9UHD8	*Homo sapiens*	Co-fractionation	[38]
***Tat***	Protein Tat	P04608	*TOR1AIP1 Hs—tat HIV-1*	Affinity Capture-MS	[27]
***TERF2***	Telomeric repeat-binding factor 2	Q15554	*Homo sapiens*	Two-hybrid	[50]
***TERF2IP***	Telomeric repeat-binding factor 2-interacting protein 1	Q9NYB0	*Homo sapiens*	Two-hybrid	[50]
***TOR1A***	Torsin-1A	O14656	*Homo sapiens*	Affinity Capture-MS/WB	[11,15,16,51,52]
				Reconstituted Complex	[52]
					[16,52]
				Affinity Capture-MS	[53]
***TOR1AIP1***	Lamina-associated polypeptide 1B	Q5JTV8	*Tor1aip1 Mm—TOR1AIP1 Hs*	Affinity Capture-MS	[35]
***TOR1B***	Torsin-1B	O14657	*Homo sapiens*	Affinity Capture-WB	[53]
***TOR2A***	Torsin-2A	Q5JU69	*Homo sapiens*	Affinity Capture-WB	[53]
***TOR3A***	Torsin-3A	Q9H497	*Homo sapiens*	Affinity Capture-WB	[53]
***UBC***	Polyubiquitin-C	P0CG48	*Homo sapiens*	Affinity Capture-MS	[54,55,56,57,58,59]
***VIM***	Vimentin	P08670	*Homo sapiens*	Affinity Capture-WB	[60]

* signifies protein-RNA and ** represents RNA-RNA interactions. Hs, Homo sapiens; Mm, Mus musculus; HRSVA, Human respiratory syncytial virus A (strain A2); HHV-4, Human herpes virus 4 (Epstein-Barr virus (strain AG876)); HIV-1, Human Immunodeficiency Virus 1; MS, mass spectrometry; WB, western blot.

The mammalian proteins’ interactions were analysed in the next section with the support of GeneMANIA.

### 2.2. Network Construction with GeneMANIA

In order to generate an extended protein-protein interaction network compiled by the set of proteins that were obtained previously, excluding RNA interactions and viral proteins, all the remaining binary interactions were imported to GeneMANIA [61]. Network mapping of LAP1 protein interactors was only possible by extrapolating the mouse and rat interactions to human. The version 3.4.0 of GeneMANIA [61] was installed into Cytoscape 3.2.1 [23] enabling network editing.

**Figure 3:**
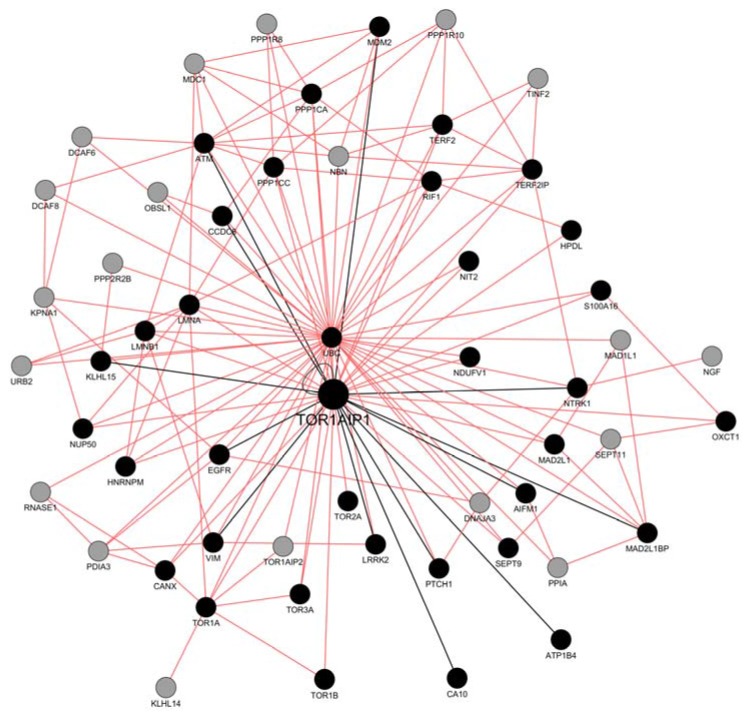
LAP1 (TOR1AIP1) interacting network complemented by the GeneMANIA plugin in cytoscape. Missing binary interactions to TOR1AIP1 were added to the GeneMANIA output and are represented by the black edges, while the remaining are in pink. Black nodes signify the interactors inserted into the plugin and grey nodes denote GeneMANIA’s additional input. LAP1 is also able to form dimers, which is represented by a self-binding edge.

The set of gene IDs was introduced and the functional associative network was set to include only physical interactions between proteins. The output includes probable protein interactors in the interactome of LAP1, extending the network based on a guilt-by-association approach that derives predictions from a combination of potentially heterogeneous data sources. Thereby, the grey nodes are genes inferred by the GeneMANIA plugin. GeneMANIA extends the user’s list with genes that are functionally similar, or have shared properties with the initial query genes, and displays an interactive functional association network, illustrating the relationships among the genes and data sets [61]. The resulting network represents a prospective LAP1 interactome (Figure 3) that we propose to have functional relevance regarding its probable integrative role as a complex assembly or stabilization protein. For instance, LAP1 association with itself could be important to form dimers that provide a structural link between the nucleoskeleton (lamina) and cytoskeleton across the NE, as previously described for SUN domain proteins in the NE (reviewed in [62]).

The association between MAD2L1 and MAD2L1BP occurs in the mitotic checkpoint complex (MCC) [63], and both proteins have been proposed to physically bind LAP1 and MAD1L1 [35] (Figure 3). The MCC inhibits the anaphase-promoting complex/cyclosome (APC/C), which is an E3 ubiquitin ligase that initiates chromosome segregation [64]. This inhibition regulates the spindle assembly checkpoint (SAC) which is responsible for delaying chromosome segregation until all sister chromatids achieve bipolar attachment to the mitotic spindle, ensuring genome stability [64]. The experimental evidence of LAP1’s direct interactions with MAD2L1 and MAD2L1BP reinforces the early hypothesis that LAP1 is a key regulator of mitosis [9,65], and strengthens the upcoming perspective that LAP1 might be functionally associated to the MCC.

Human LAP1B was previously described to be phosphorylated on Ser143, in a proline-directed manner similar to CDKs substrates [66], and on Ser164 within the ATM/ATR recognition sequence motifs [67]. Furthermore, rat LAP1B Ser142 (homologous to human LAP1B Ser143) was found to be phosphorylated by CDK1-cyclin B [68]. Indeed, LAP1 seems to be functionally associated with several signalling proteins that activate, inhibit and mediate signalling cascades. Among the most relevant regulators are protein kinases, in this case comprising EGFR, NTRK1, LRRK2 and ATM (Figure 3). DNA damage events, such as ultraviolet irradiation, result in the nuclear translocation of EGFR, in a ligand-independent pathway [69]. Similarly, LAP1 phosphorylation by ATM on S133, S145, S169 and S164 was associated to DNA damage, consolidating the hypothesis that LAP1 might be phosphorylated as a cellular response to genotoxic stress [32].

Alternatively, protein phosphatases PP1α (*PPP1CA*) and PP1γ (*PPP1CC*) were shown to regulate LAP1 by dephosphorylation, forming a complex that was validated in rat brain and cultured cells [19]. This reveals the role of LAP1 in complexes important in regulation, through phosphorylation. Moreover, the PP1 regulatory protein CCDC8 (alternatively termed PPP1R20) is a core component of the 3-M complex. This complex is required to regulate microtubule dynamics and genome integrity [36]. Correspondingly, CCDC8 associates with the mRNA splicing machinery, in particular to the HNRNP family, which is highly represented in the 3-M interactome [36]. The above-mentioned LAP1 mediated events illustrate, the potential dynamic role of LAP1, in the regulation of transcription and cytoskeleton mechanisms via interaction with CCDC8 (Figure 3).

LAP1 might communicate to the shelterin complex. The latter is an emerging protein complex with DNA remodelling activity that coordinates with DNA repair factors, to change the structure of the telomeric DNA, thereby protecting chromosome ends (reviewed in [70]). This complex is composed of TRF1 and TRF2 as double-stranded DNA binding proteins that recognize TTAGGG repeats [50]. TRF2 in particular is associated to TERF2IP which is then regulated by RIF1 (reviewed in [70]). RIF1 is required for checkpoint mediated arrest in response to DNA damage during the *S*-phase (the intra-*S*-phase checkpoint) [71]. This checkpoint can be activated by at least by two parallel pathways involving the ATM kinase [71]. LAP1 might have a role in assembly or stabilizing this particular subset of proteins of the shelterin complex, as it binds to TRF2, TERF2IP, RIF1 and ATM (Figure 3). Telomere associated functions have been previously described for various INM proteins such as LMNA, SUN1, LAP1 and BAF (reviewed in [72]).

The relevance of LAP1 interactions regarding biological processes and cellular localizations were analysed in the next section with the support of GO enrichment analysis [73] and BiNGO [74].

### 2.3. GO Term Enrichment Analysis

**Figure 4:**
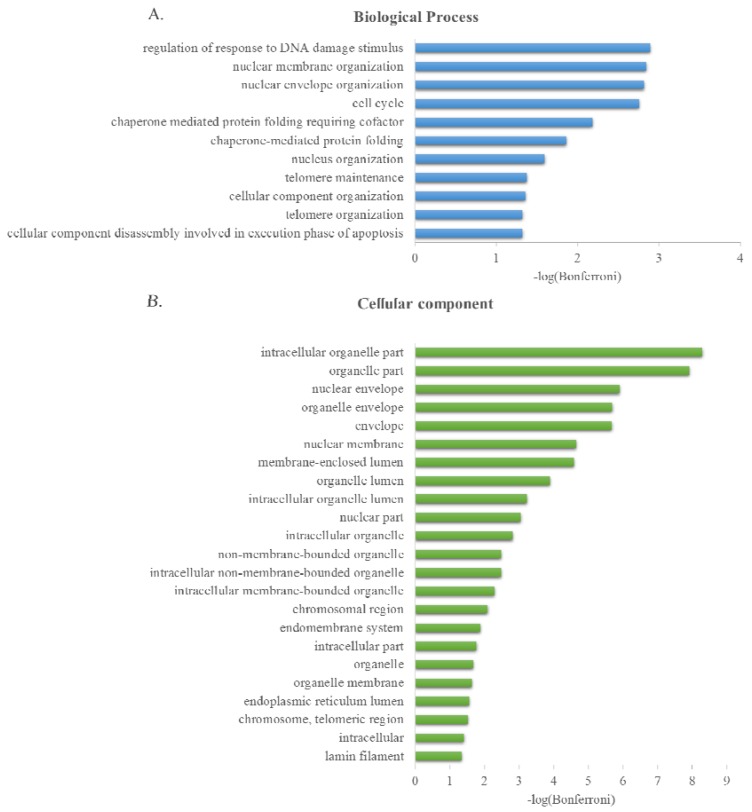
Significantly enriched Gene Ontology terms from LAP1 interaction network (**A**) Biological process; and (**B**) Cellular Component. Bonferroni corrected *p*-values (α = 0.05) were transformed by −log10.

The online GO Consortium term enrichment service, supported by Panther (available at [75]) [73,76], was used to conduct a GO term enrichment analysis for the 38 LAP1 interactors described in Table 1. Viral connections (tat, 1C and LMP2) were excluded. The *biological process* and *cellular component* GO terms that were enriched among these target proteins were scored (Figure 4). All the IDs of the interactors were mapped with one exception, SCARNA22.

BiNGO [74] was alternatively used to build a directed acyclic graph (DAG) network that conveys visualization of the enriched terms organized in a tree-like structure, starting from more general terms at the root (for example, biological regulation) to the most specific at the leaves (for example, the regulation of mitotic cell cycle) [74]. Each term is coded by size and colour, so that larger nodes contain more genes and darker nodes are more significantly enriched. Insignificant intermediate terms are denoted as small white nodes [74] (Figures S1 and S2).

Upon examining the biological processes of the proteins found in the filtered list of interactions (Figure 4A and Figure S1), significant enrichment was observed in the processes of “regulation of response to DNA damage stimulus”, “nuclear membrane organization”, “nuclear envelope organization”, “cell cycle”, “chaperone mediated protein folding requiring cofactor”, “chaperone-mediated protein folding”, “nucleus organization”, “telomere maintenance”, “cellular component organization”, “telomere organization” and “cellular component disassembly involved in execution phase of apoptosis”. By analysing the cellular component that the protein interactors might integrate (Figure 4B and Figure S2), significant and relevant enrichment was found in the “nuclear envelope”, “chromosomal region”, “endoplasmic reticulum lumen”, “chromosome and telomeric region” and “lamin filament”.

This output reinforces the idea that LAP1 might function as a stabilizing element by forming multiple complexes as noted before. In particular, integrating a response to DNA damage through ATM activation and subsequently participating in telomere regulation, through the interaction with RIF1, TERF2IP and TRF2 [71]. Furthermore, there are strong evidences that LAP1 intervenes in mitotic regulation via association to the MCC complex proteins, as previously studied [9]. The vastly documented relationship with the Torsin family [77], resident in the endoplasmic reticulum, conveys the chaperone related annotations observed in the enrichment analysis. Finally, LAP1 collaboration with the NE cytoskeleton [9,77] outputs “nuclear membrane organization”, “nuclear envelope organization”, “cellular component organization” and “nucleus organization”, as some of the most prominent biological processes attributed to LAP1 interactors.

Physiological and functional analysis of the pathways’ with which LAP1’s interactome integrates is presented in the next section, with the aid of the Ingenuity Pathway Analysis.

### 2.4. Ingenuity Pathways Analysis (IPA) Physiological and Functional Analysis

**Figure 5:**
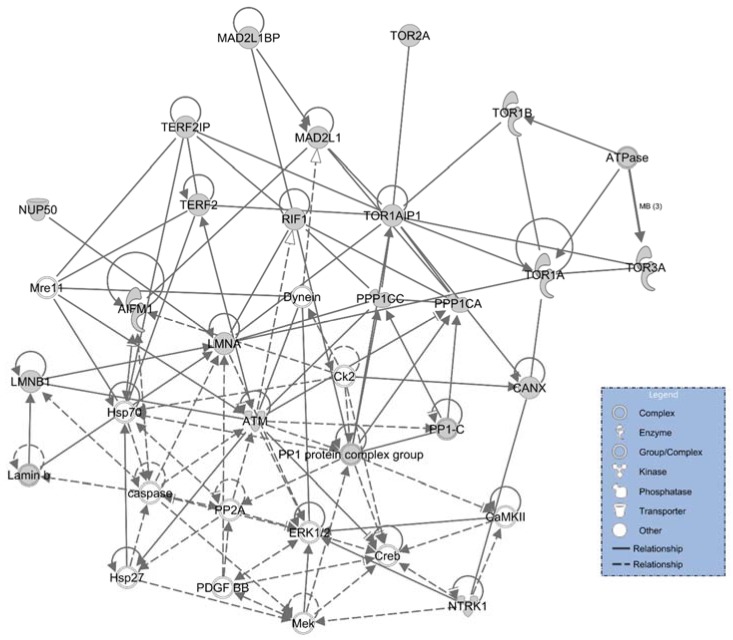
Network ID 1. IPA Associated Network Functions: Cell Morphology, Cellular Assembly and Organization, DNA Replication, Recombination, and Repair. Grey nodes represent proteins that are integrated in the initial dataset, while white nodes convey IPA’s supplementary missing proteins.

Gene identifications of the previously described interactors were imported into the Ingenuity Pathways Analysis (IPA) tool, once more extrapolating the mouse and rat interactions to human and excluding viral connections. Function classifications, signal pathways and interacting networks were constructed and analysed based on the underlying biological evidence from the IPA’s database. The core analysis performed in IPA retrieved seven distinctive functional sets: canonical pathways, upstream regulators, diseases and disorders, molecular and cellular functions, physiological system development and functions, associated network functions and toxicological lists that are depicted in Table 2, Table 3, Table 4, Table 5 and Table 6 and Figure 5, Figures S3–S6. Right-tailed Fisher’s exact test was used to calculate the significance of each biological function and/or disease assigned, which was reported if the adjusted *p*-value was below a significance threshold of 0.05.

**Table 2:** Top Canonical Pathways for LAP1 interactors dataset.

Name	*p*-Value	Overlap %	
Telomerase Signalling	2.99 × 10^−5^	4.00%	4/99
Telomere Extension by Telomerase	3.28 × 10^−4^	13.30%	2/15
HER-2 Signalling in Breast Cancer	3.47 × 10^−4^	3.90%	3/76
Glioma Signalling	6.68 × 10^−4^	3.20%	3/95
Huntington’s Disease Signalling	7.49 × 10^−4^	1.70%	4/229

*Overlap* represents the percentage of LAP1 interactors integrated in the total number of proteins associated to each specific pathway on the IPA Knowledge Base.

**Table 3:** Physiological System Development and Function for LAP1 interactors dataset.

Name	*p*-Value	# Molecules
Skeletal and Muscular System Development and Function	3.61 × 10^−3^–1.82 × 10^−6^	7
Tissue Development	3.61 × 10^−3^–1.82 ×10^−6^	13
Nervous System Development and Function	3.61 × 10^−3^–3.93 × 10^−6^	13
Organ Morphology	3.61 × 10^−3^–3.93 × 10^−6^	9
Tissue Morphology	3.61 × 10^−3^–3.93 × 10^−6^	16

**Table 4:** Top Associated Networks for LAP1 interactors dataset.

ID	Associated Network Functions	Score
**1**	Cell Morphology, Cellular Assembly and Organization, DNA Replication, Recombination, and Repair	47
**2**	Cancer, Organismal Injury and Abnormalities, Respiratory Disease	32
**3**	RNA Post-Transcriptional Modification, Protein Synthesis, Gene Expression	3
**4**	Developmental Disorder, Neurological Disease, Behaviour	2

*Score* attributes a numerical value used to rank networks according to how relevant they are to the genes in the input dataset.

**Table 5:** Top Toxicological Lists for LAP1 interactors dataset.

Name	*p*-Value	Overlap %
Hypoxia-Inducible Factor Signalling	2.72 ×10^−4^	4.3% 3/70
Mitochondrial Dysfunction	3.90 × 10^−3^	1.7% 3/176
Cell Cycle: G2/M DNA Damage Checkpoint Regulation	3.97 × 10^−3^	3.8% 2/52
Cell Cycle: G1/S Checkpoint Regulation	6.32 × 10^−3^	3.0% 2/66
TR/RXR Activation	1.03 × 10^−2^	2.4% 2/85

*Overlap* represents the percentage of LAP1 interactors integrated in the total number of proteins associated to each specific pathway on the IPA Knowledge Base.

**Table 6:** Molecular and cellular functions for LAP1 interactors dataset.

Name	*p*-Value	# Molecules
Cell Morphology	3.61 × 10^−3^–3.18 × 10^−11^	18
Cellular Assembly and Organization	3.61 × 10^−3^–3.18 × 10^−11^	20
DNA Replication, Recombination, and Repair	3.61 × 10^−3^–1.34 × 10^−10^	15
Cell Cycle	3.61 × 10^−3^–9.17 × 10^−9^	17
Cell Death and Survival	3.61 × 10^−3^–4.21 × 10^−7^	20

IPA reinforced the idea that LAP1 might be communicating with the shelterin complex, as is evident in Table 2, where the top two canonical pathways include “Telomerase signalling” and “Telomere Extension by Telomerase”. Furthermore, IPA analysis of physiological system development and function identified “Skeletal and Muscular System Development and Function” as the most relevant (Table 3). This result is in accordance with the previous described functions of LAP1 regarding its association with the Torsin family [77].

Regarding the rank for associated networks in Table 4, the analysis is not limited to molecular interactions. The result delivered the most pertinent functions of LAP1 interactors, which is attributed to “Cell Morphology, Cellular Assembly and Organization, DNA Replication, Recombination, and Repair” (Figure 5).

Additionally, IPA retrieved BRAC1 as the most probable upstream regulator of the LAP1 interactome (Figure S3). This inference is in accordance with the regulation of important players in the proposed network for LAP1 interactors (Figure S3), namely EGRF and ATM concerning DNA damage events and MAD2L1, which might be activated for mitosis regulation with the MCC. Furthermore, the data shown in Table 5 also confirms this hypothesis, with the identification of “Cell Cycle: G2/M DNA Damage Checkpoint Regulation” and “Cell Cycle: G1/S Checkpoint Regulation” as top toxicological attributes.

Lastly, IPA’s analysis of molecular and cellular functions attributed to LAP1’s interactome (Table 6) acknowledges “Cell Morphology” and “Cellular Assembly and Organization” as the most pertinent, followed by “DNA Replication, Recombination, and Repair”, “Cell Cycle” and “Cell Death and Survival”. The networks describing and integrating these functions are available in the Supplementary Figures S4 and S5. By studying these interactions it is possible to deduce the interconnected pathways in which LAP1 might participate, namely, suggesting a crucial role in regulating nuclear morphology, cell cycle progression and cell survival.

Ingenuity pathway analysis confirmed the hypothesis that LAP1, besides the functional associations with lamins and nuclear morphology, might be a crucial player in cell cycle progression and DNA damage responses. These conclusions are in accordance with the prior analysis performed with GO and GeneMANIA.

## 3. Experimental Section

### 3.1. Collection of Associated Interactions of LAP1

In order to develop a comprehensive computational analysis of the LAP1 interactome, experimentally detected protein-protein and protein-nucleic acid interactions were extracted from the following public databases: BioGRID [78] (version 3.4.129, consulted 12 October 2015), IntAct [79] (version 4.1.8, consulted 13 October 2015), mentha [80] (version of 13 October 2015I2D [81] (version of 13 October 2015), APID[82] (version of 13 October 2015), HIPPIE [83] (version of 13 October 2015), NCBI [84] (version of 13 October 2015). Additionally, the PSICQUIC web service [85] (version 1.4.5, consulted 14 October 2015) enabled access to multiple PSI-MI compliant resources.

The resulting set of 180 binary interactions includes high and low-throughput data that was later manually curated by consulting the corresponding articles. Despite the worth and accessibility of protein interaction data from online databases, it also comes with limitations. Numerous databases (including the ones used in this analysis) contain interactions based on indirect evidence, for instance, data mining, genetic interactions, metabolic evidence and co-localization data. In this case, the gene set had a dimension that enabled the manual confirmation of each binary interaction. However, when the protein of interest retrieves a greater number of interactors, individually validating the curated interactions, of the results obtained might not be feasible. In these cases, filtering on the website enables the authors to clean the data prior to export. Alternatively, it is possible to import PSI-MI XML or XGMML into Cytoscape and use the filtering tools available. The output from our approach resulted in the identification of 41 LAP1 interactors (Table 1 and Figure 2).

### 3.2. Gene Ontology Term Enrichment Analysis

Gene Ontology (GO) provides a system of terms to consistently describe and annotate gene products [86]. GO term enrichment analysis (supported by Panther 10.0, released 15 May 2015; analysed 15 October 2015) [76] was performed online using the *Search GO data* tool (available at [75]) [73] to enquire for GO terms that are over-represented using the annotations regarding *biological process* and *cellular component*. The list of genes was pasted into the corresponding box to be analysed, choosing *Homo sapiens* as the species of genes integrated in the gene set. GO analysis of LAP1 the interactome was only possible by extrapolating the mouse and rat interactions to human. The output retrieved a table that lists significant shared GO terms (or parents of GO terms) used to describe the set of genes that users entered on the previous page, the background frequency, the sample frequency, expected *p*-value, an indication of over/underrepresentation for each term, and *p*-value. The statistical analysis included Bonferroni’s correction for multiple testing and a term was reported as enriched if the adjusted *p*-value is below a significance threshold of 0.05. All the input genes were mapped, however two were unclassified: TOR3A for biological process ontology and HPDL for cellular component. The *p*-values were corrected for multiple testing using the Bonferroni procedure and transformed by taking the -log10 for easier visualization. The online GO term enrichment service, retrieved from the online platform was stored and analysed as Excel spreadsheet files (Figure 4).

### 3.3. Software Platforms and Plugins

Data obtained from the online databases already described were stored and analysed as Excel spreadsheet files. The network of LAP1 interactors was built using Cytoscape (3.2.1, freely available online at [87]) [23]. Cytoscape is one of the most popular tools for visualizing, integrating, modelling, and analysing molecular and genetic interaction networks [23].

The GeneMANIA plugin (version 3.4.0) was installed using the Cytoscape App Manager (Cytopscape plugin can be download at [88]) [61]. GeneMANIA is a particularly convenient and user-friendly metasearch platform that generates a highly correlated signalling network centred on the proteins of interest [89]. To determine the interactions between the LAP1 interactors inside the network, the gene set was introduced into GeneMANIA on Cytoscape. Different results can be produced from the same gene list when using GeneMANIA. In this case, by only selecting *physical interaction* data, the network will only show genes linked if the proteins in question share a binary interaction established through physical experiments [89]. The integrated network obtained after using GeneMANIA was subsequently manually validated for curated interactions with the respective publications, to confirm the binary interactions. With the editing tools offered by Cytoscape this network was designed to obtain the final result presented in Figure 3.

BiNGO is a tool used to determine which Gene Ontology (GO) categories are statistically overrepresented in a set of genes and outputs a network that allows interactive visualization of results mapped by the GO hierarchy [74]. The BiNGO plugin (version 3.0.3) was installed using the Cytoscape App Manager (freely available to download at [90]) [74]. In Cytoscape, BiNGO outputs a network of the significant GO terms using *p-*values that were corrected for multiple testing using the Bonferroni correction. Each term is size and colour-coded, so that larger nodes have more genes and darker nodes are more significantly enriched. Intermediate terms that are not significant are present as small white nodes [74]. GO terms are organized in a tree-like structure, starting from more general terms at the root (for example, biological regulation) to the most specific at the leaves (for example, the regulation of mitotic cell cycle) distributed across the two main semantic domains—*biological process* and *cellular location*. As GO terms might have more than one parent, they are technically structured as a network called a DAG (Figures S1 and S2).

Ingenuity^®^ Pathway Analysis (IPA^®^, QIAGEN Redwood City,[91]) is a commercially available software program that helps model, analyse and understand complex biological and chemical systems. The Ingenuity Knowledge Base is a repository of expertly curated biological information. Gene identifications of the previously described interactors were imported into the Ingenuity Pathways Analysis (IPA) tool, once more extrapolating the mouse and rat interactions to human and excluding viral connections. Function classifications, signal pathways and interacting networks were constructed and analysed based on the underlying biological evidence from the IPA’s database. The core analysis performed in IPA retrieved seven distinctive functional sets: canonical pathways, upstream regulators, diseases and disorders, molecular and cellular functions, physiological system development and functions, associated network functions and toxicological lists. The output generated through the use of QIAGEN’s Ingenuity Pathway Analysis (IPA^®^, QIAGEN Redwood City, CA, USA [91]) is depicted in Table 2, Table 3, Table 4 and Table 5 and Figure 5, Figures S3–S6. Right-tailed Fisher’s exact test was used to calculate the significance of each biological function and/or disease assigned, which was reported if the adjusted *p*-value was below a significance threshold of 0.05.

## 4. Conclusions

The integration of multiple bioinformatic tools allowed for the development of an intricate study of LAP1 interactors. These analyses attribute functions to LAP1 that have not been previously described. For instance, various DNA damage response proteins that incorporate or regulate the shelterin complex were shown to directly interact with LAP1. This function has been previously associated with INM proteins such as LMNA, SUN1, LAP1 and BAF. Additionally, the previously described association of LAP1 with mitosis was confirmed through the interaction with MCC. Therefore, these and other prospective functions of LAP1 convey the significant role of INM proteins in the regulation of multiple cellular processes and disease conditions.

## Supplementary Materials

Figure S1: Significantly enriched Gene Ontology terms from the LAP1 interaction network concerning Biological Processes. Figure S2: Significantly enriched Gene Ontology terms from the LAP1 interaction network concerning Cell Components. Figure S3: Top Upstream Regulators of the LAP1 interactome. Figure S4: Molecular and cellular functions for LAP1 interactors. Figure S5: Merged molecular and cellular functions associated with LAP1 interactors.
