# Supplementary material for: Lamina Associated Polypeptide 1 (LAP1) Interactome and Its Functional Features
Source: Membranes (Basel). 2016 Jan 15;6(1):8. doi: 10.3390/membranes6010008 (PMC4812414; doi:10.3390/membranes6010008)
Supplement: Supplementary file 1 [file membranes-06-00008-s001.docx]

Supplementary Materials: Lamina Associated Polypeptide 1 (LAP1) Interactome and Its Functional Features

Joana B. Serrano, Odete A. B. da Cruz e Silva and Sandra Rebelo


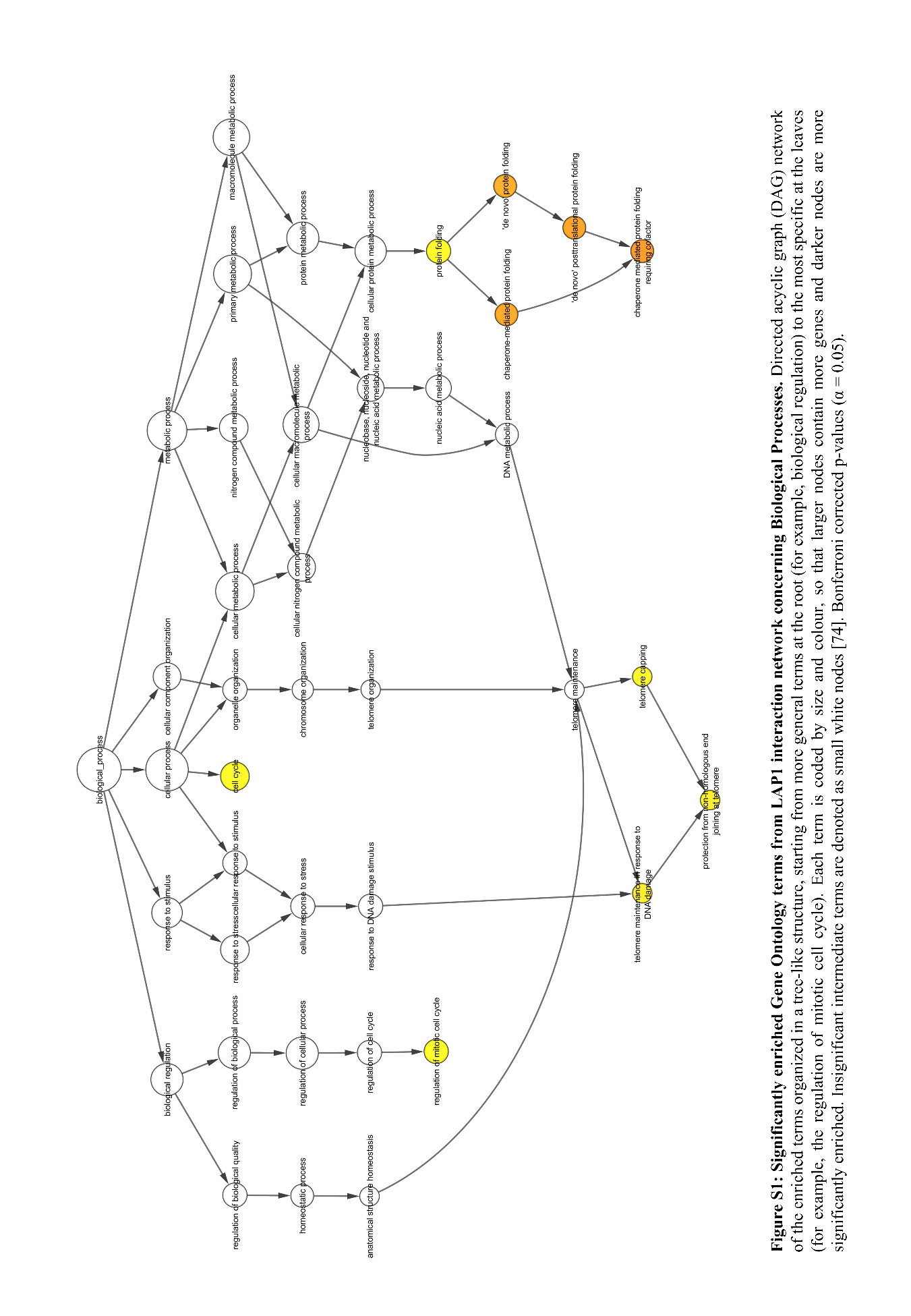


**Figure S1.** **Significantly enriched Gene Ontology terms from LAP1 interaction network concerning Biological Processes.** Directed acyclic graph (DAG) network of the enriched terms organized in a tree-like structure, starting from more general terms at the root (for example, biological regulation) to the most specific at the leaves (for example, the regulations of mitotic cell cycle) [72]. Each term is coded by size and colour, so that larger nodes contain more genes and darker nodes are more significantly enriched. Insignificant intermediate terms are demoted as small white nodes [72]. Bonferroni corrected *p*-values (α = 0.05)


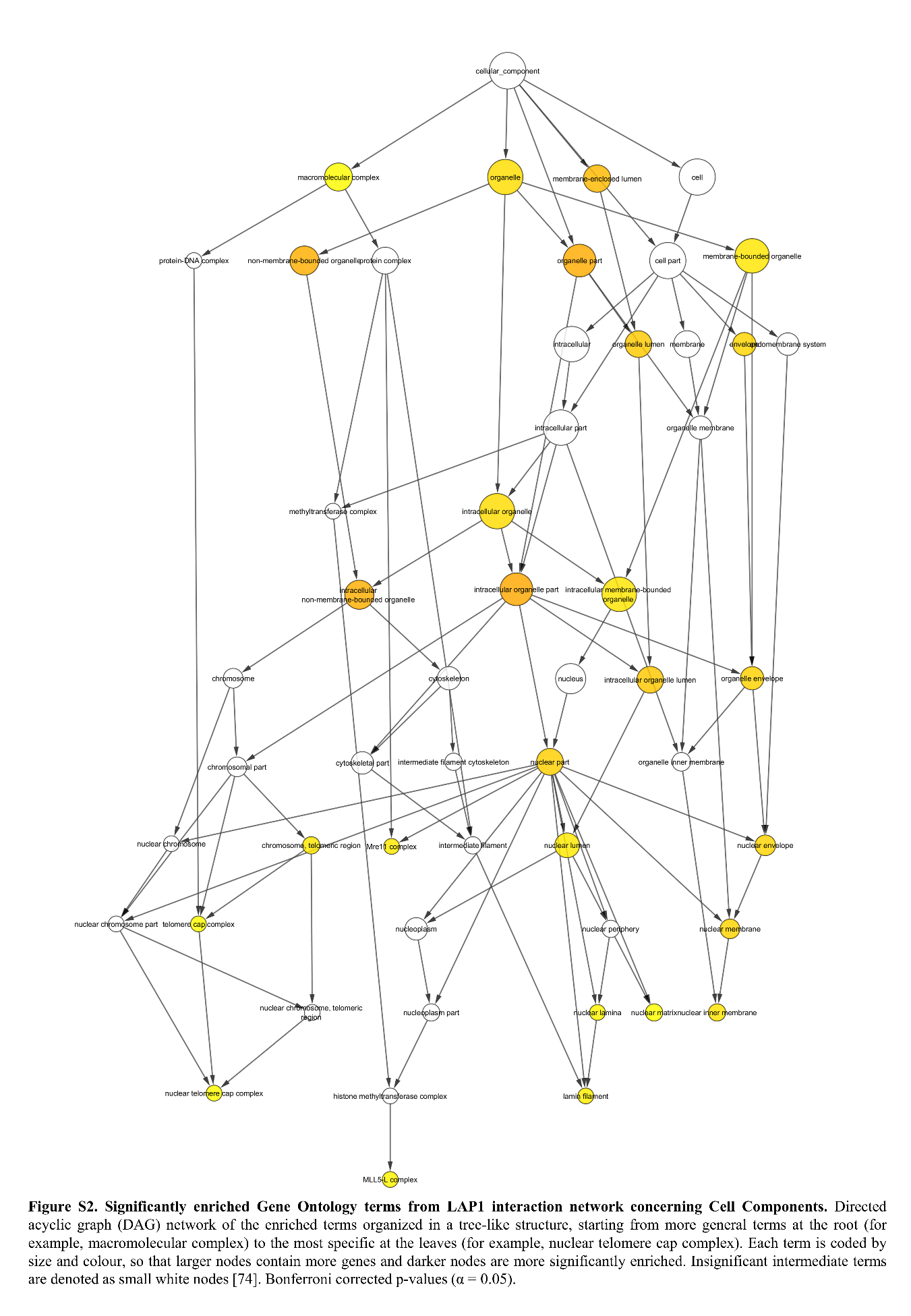


**Figure S2.** **Significantly enriched Gene Ontology terms from LAP1 interaction network concerning Cell Components.** Directed acyclic graph (DAG) network of the enriched terms organized in a tree-like structure starting from more general terms at the root (for example, macromolecular complex) to the most specific at the leaves (for example, nuclear telomere cap complex) [72]. Each term is coded by size and colour, so that larger nodes contain more genes and darker nodes are more significantly enriched. Insignificant intermediate terms are demoted as small white nodes [72]. Bonferroni corrected *p*-values (α = 0.05)


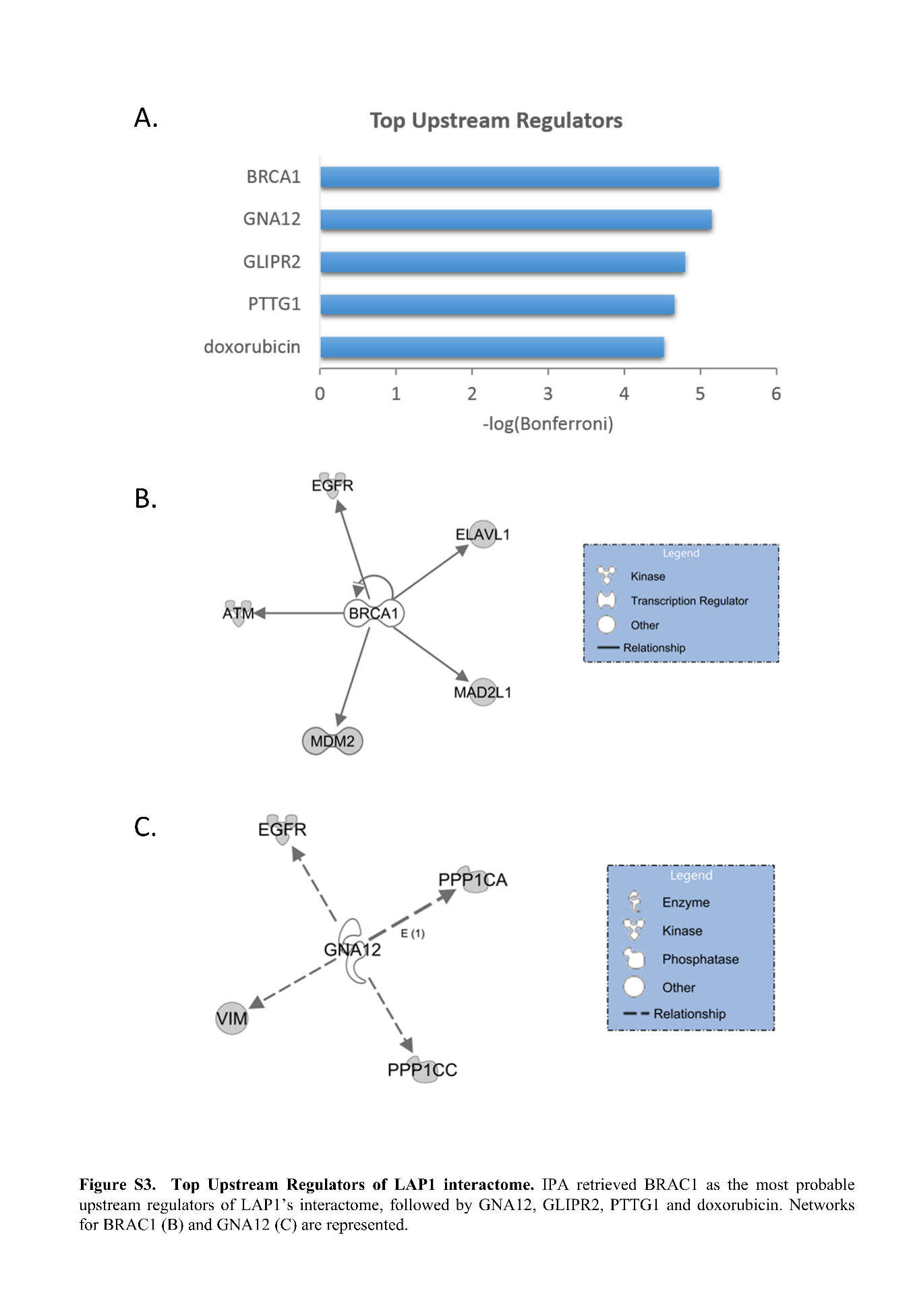


**Figure S3.** **Top Regulators of LAP1 interactome.** IPA retrieved BRAC1 as the most probable upstream regulators of LAP1’s interactome, followed by GNA12, GLIPR2, PTTG1 and doxorubicin. Networks for BRAC1 (**B**) and GNA12 (**C**) are represented.


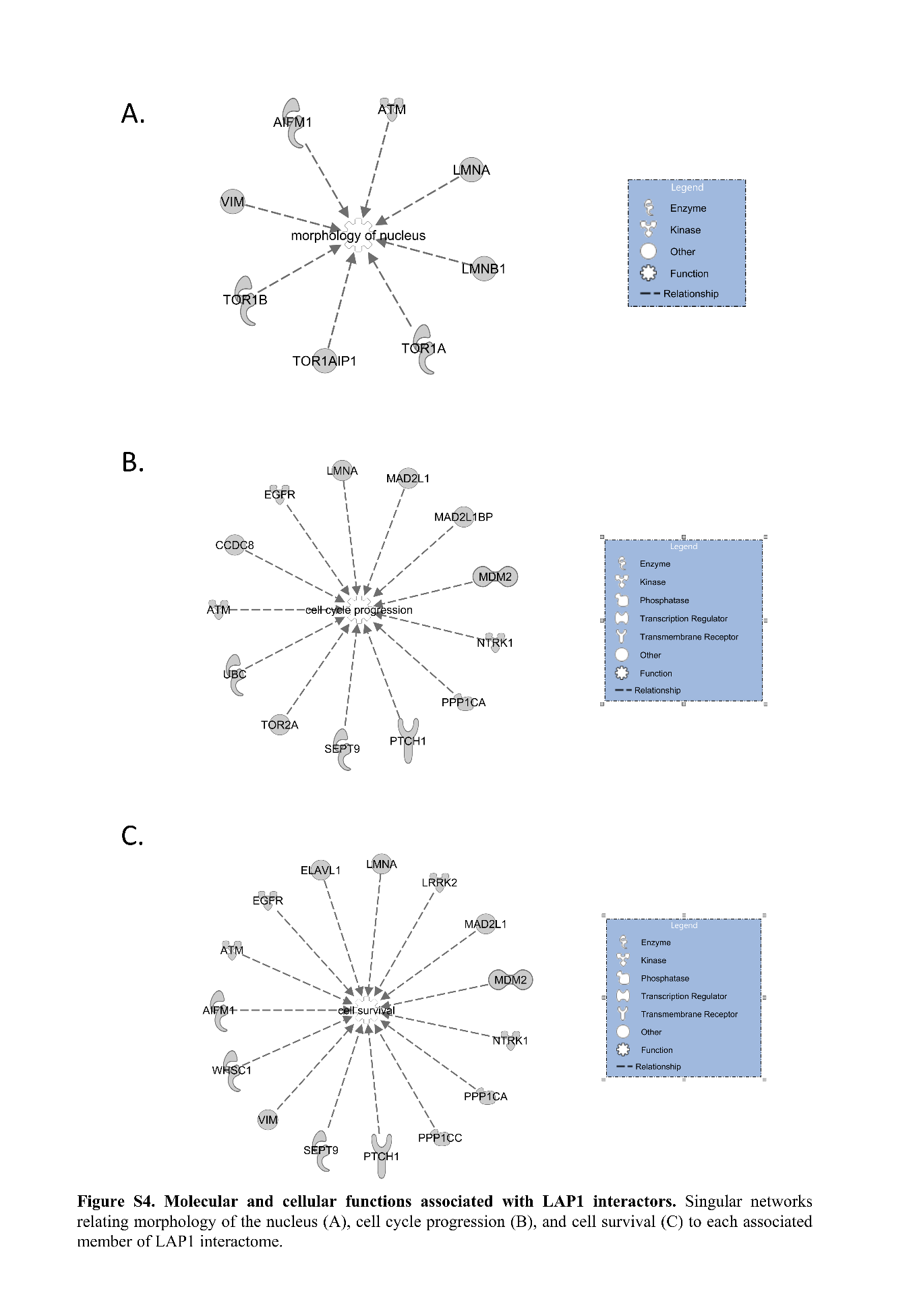


**Figure S4.** **Molecular and Cellular functions associated with LAP1 interactors.** Singular networks relating morpholpogy of the nucleus (**A**); cell cycle progression (**B**); and cell survival (**C**) to each associated member of LAP1 interactome.


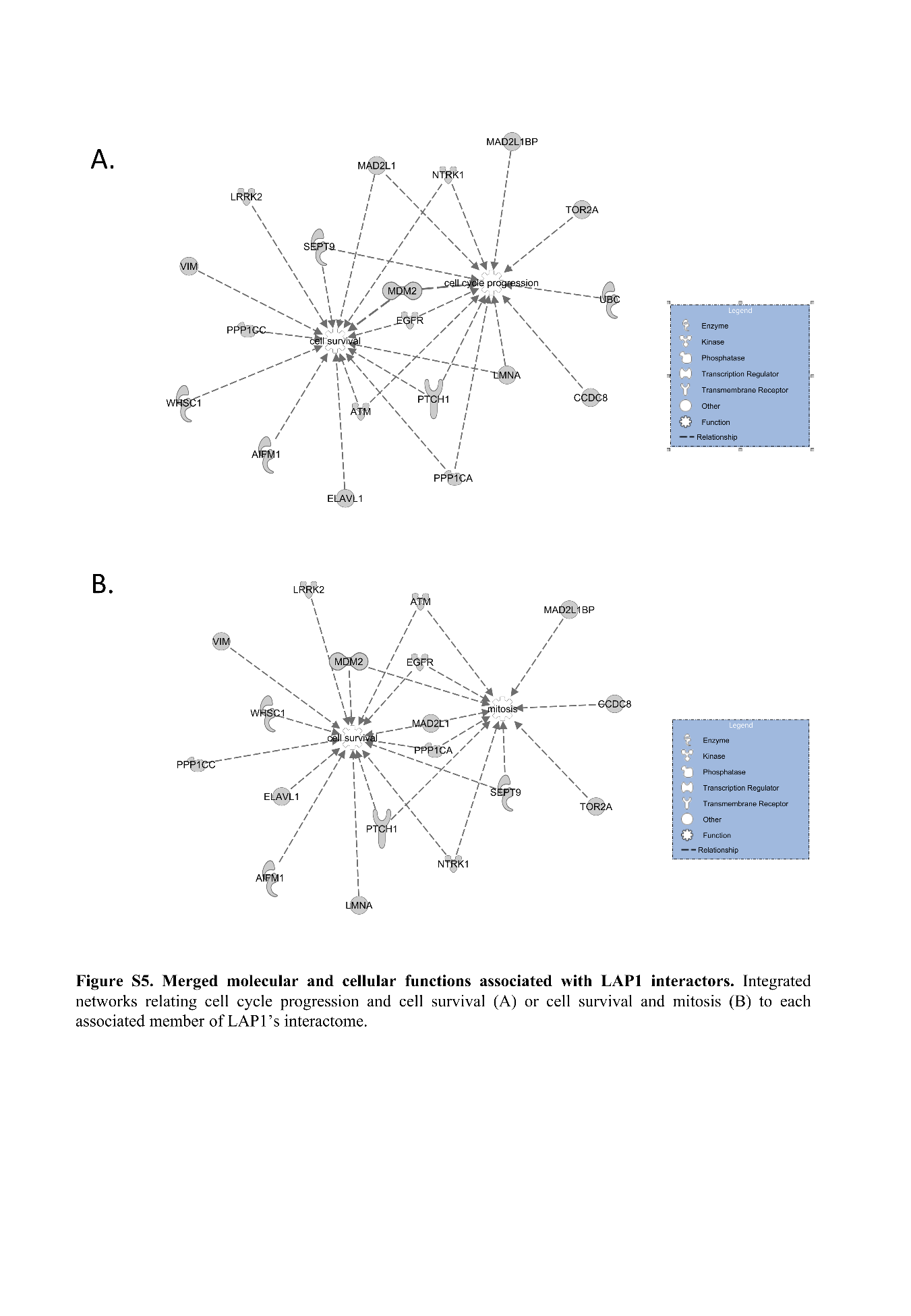


**Figure S5.** **Merged molecular and cellular functions associated with LAP1 interactors.** Integrated networks relating cell cycle progression and cell survival (A) or cell survival and mitosis (B) to each associated member of LAP1’s interactome.
